# 

**Published:** 1899-11

**Authors:** 


### CONTENTS:

### SELECTIONS.

ARTICLE I.—Brittle Platinum Pins in Porcelain Teeth. George M. P. Murray, F. R. C. S .......... 289
" II.—The Influence of the Quality of Drinking Water in the Structure of the Teeth. M. E. Louguet .......... 294
" III.—Bacteriology in the Treatment of Disease C. T. Whinery, D. D. SC .......... 296
" IV.—Nervanin. Dr. A. D. Kyner .......... 300
" V.—The Value of High Fusing Porcelain in Contour Work. Joseph Head, M. D.. D. D. S .......... 301
" VI.—Suitable Recreations for Dentists. M Cavanagh, D. D. S .......... 309
" VII.—Cocainism. J. Harrington Beynon. M. D .......... 321
" VIII.—Dental Jusisprudence .......... 324
" IX.—"The Outside of the Platter." .......... 329

### OBITUARX.

W. G. A. Bonwill Dead .......... 331

### MONTHLY SUMMARY.

Combination Fillings .......... 332

Simple Test for Good Plaster .......... 332

Varnishing Cavities .......... 333

Succesgful Amputation of Pulp .......... 333

Cement .......... 334

"Waterproof" Cements .......... 335

An Alkalin Wash .......... 335

Steel Wire in the Mouth .......... 336

To Dam a Molar .......... 336

Broken Broach .......... 336

